# "Bacterial Meningitis in children and adolescents: an observational study based on the national surveillance system"

**DOI:** 10.1186/1471-2334-5-103

**Published:** 2005-11-15

**Authors:** Félix O Dickinson, Antonio E Pérez

**Affiliations:** 1 Department of epidemiology, Institute of Tropical Medicine "Pedro Kourí", Havana, Cuba; 2 Autopista Novia del Mediodía Km. 6 Municipio Lisa, Ciudad de La Habana, Cuba. P.O. Box 601 Marianao 13, Cuba

## Abstract

**Background:**

Bacterial meningitis is a group of life threatening infections that mostly affect children and adolescents, and may be the cause of severe neurological sequelae. Cuba has implemented massive vaccination programmes against both *Neisseria meningitidis *(serogroup C in 1979 and B in 1987), and *Haemophilus influenzae *type b (1999), two of the main causal pathogens. We described and discussed some epidemiological aspects of the current status of bacterial meningitis to learn from the Cuban experience.

**Methods:**

A nationwide observational study on children and adolescents from 1 to 18 years old was carried out from 1998 to 2003, estimating the incidence and case-fatality rate by age group and causal pathogens, as well as the seasonality and frequency of overcrowded dormitories. The association between disease and attendance to day care centres or boarding schools was estimated by using relative risk (Chi-squared test and Fisher Exact Test).

**Results:**

The overall number of cases was 1023; the incidence ranged from 3.4 to 8.5 per 100 000 population, with the higher figures in children 1–5 years old (16.8 per 100 000 population). *Streptococcus pneumoniae*, *Haemophilus influenzae *type b and *Neisseria meningitidis *serogroup B were the main identified agents. The average case-fatality rate was 10.5% and the most lethal agents were *Streptococcus pneumoniae *(27%) and *Haemophilus influenzae *type b (10.7%). Overall percentage of cases who slept in overcrowded dormitories was 15%, reaching 30.6% in adolescents. Seasonality was only evident among meningococcal meningitis cases between September–October. The attendance to boarding high school showed an association with disease only in 1998 and 1999 (RR = 2.1; p > 0.05).

**Conclusion:**

The highest incidence of bacterial meningitis was observed among children from 1–5 years old. Pneumococcus was both the leading causal and the most lethal agent. Sleeping in overcrowded dormitories was more frequent among adolescents. No strong association was observed between the bacterial meningitis and attendance to day care centres or boarding schools. The incidence of bacterial meningitis in Cuba is declining after massive vaccination programmes against *Neisseria meningitidis *serogroup B and C and *Haemophilus influenzae *type b through a national immunisation program.

## Background

Bacterial meningitis (BM) is a group of severe infections that frequently affect children and adolescents, carrying a high case-fatality rate (CFR). It may cause transient or permanent deafness as well as other severe neurological sequelae in survivors [1-3].

The World Health Organisation estimated that annually BM causes at least 1.2 million cases worldwide and of those 135 000 deaths. Though other bacteria may cause BM, *Neisseria meningitidis*, *Haemophilus influenzae *type b (Hib) and *Streptococcus pneumoniae *(Spn) are the main triad responsible for more than 80% of all cases. Gram negative bacteria, particularly *E. coli*, *Streptococci *(different from *S. pneumoniae*), *Listeria monocitogenes *and *Staphylococci *may also cause BM [3].

BM has been reported in Cuba since the beginning of the 20^th ^century [4]. The mandatory report of this group of infections has been implemented since 1961 as part of national communicable diseases surveillance.

Following a meningococcal disease epidemic in 1979, an independent surveillance system was put into place [4]. After controlling the epidemic, other germs like Hib and Pneumococcus increased their aetiological importance. In 1997 public health authorities decided to extend the surveillance to all bacteria, as well as to improve epidemiological and microbiological data through the Bacterial Meningitis National Surveillance System (BMSS) [5]. This allowed a high level of accuracy and quality information to be collected which could be compared with high surveillance systems international standards [5-8].

On the other hand, Cuba is the only country in the world which has carried out massive vaccination initiatives against *Neisseria meningitidis *serogroup C (1979) and B (1987) [9], and later in 1999 against Hib [10], both as part of the national immunisation program (NIP).

The aim of this study was to describe and discuss the results of both, the BMSS and the massive vaccination strategies, on the epidemiology of BM in Cuban children and adolescents.

## Methods

1023 cases of BM in the Cuban population between 1–18 years old reported nationwide by BMSS from January 1^st ^1998 to December 31^st ^2003 were included in the study, considering the date of the symptoms onset.

Children under 1 years old were not included in the study based on the criteria that they should be considered a special group for the following reasons: breast-feeding, immune maturation, they usually do not attend day care and do not have much community contact.

We defined a case of BM as "a clinical meningeal syndrome, through the identification of the causal agent directly by culture blood, petecchias, cerebrospinal fluid (CSF) analysis or indirectly by polymerase chain reaction, latex test or another rapid diagnostic test. The cases with negative culture or bacteriological test, but the cyto-chemical exam of the CSF suggested bacterial infection, were considered BM of "unknown aetiology" [11].

A total of 4 age groups which coincided with the main level of education in Cuba were selected: the group 1–5 year olds which attended day care centres (DCC) was considered as small children; 6–11 year olds were considered older children (Primary School). More than 11 year olds were considered adolescents, attending High School those 12–14 year olds and to College, Technical or Professional Education those 15–18 year olds.

The incidence rate (new cases per 100 000 population) and CFR (percent) were calculated according to the age group and to the main causal agent, using the data of estimated Cuban population of the Office for National Statistics in Cuba. The seasonality by monthly average for each causal agent and the frequency of cases who slept in overcrowded dormitories by age group were also estimated. Comparisons were carried out using Chi-squared tests with a significance level of 0.05.

Relative Risk (RR) was estimated and its confidence interval with p < 0.05 considered as significant, using either a Chi-squared Test or Fisher's Exact Test as appropriate. We considered exposed to community settings the cases attending DCC or boarding school, and not exposed those who do not attend these institutions. The national matriculation and registration of school children and adolescents during each school year were obtained from the Ministry of Education (MINED). RR in primary school children was not estimated because the boarding student matriculate between 1998–2003 was not available.

We used Epi-Info (version 6.2^a^) and Excel (version 5.1) for the statistical analysis, and Microsoft Word 2000 as the text processor program.

## Results

A total of 1023 cases of BM between 1998–2003 were reported. The overall incidence rose significantly from 6.5 per 100 000 population in 1998 to 8.5 per 100 000 population in 1999, but it subsequently decreased to 4.2 per 100 000 population in 2003 (Table 1).

**Table 1:** Overall Incidence and Case-Fatality Rate (CFR) for bacterial meningitis* by age group in Cuba from 1998–2003.

**Age group**	**1998**	**1999**	**2000**	**2001**	**2002**	**2003**
1–5 yrs	16.8	15.3	11.1	8.5	4.7	6.2
6–11 yrs	3.3	7.4	4.1	3.7	3.3	4.6
12–14 yrs	1.6	5.9	3.7	3.5	4.3	4.1
15–18 yrs	3.7	4.5	4.5	2.9	1.7	2.0

**Overall Incidence**	**6.5**	**8.5**	**5.8**	**4.6**	**3.4**	**4.2**

**Overall CFR**	**7.8**	**9.0**	**13.6**	**13.6**	**8.7**	**10.9**

* Bacterial meningitis caused by *Streptococcus pneumoniae*, *Neisseria meningitidis*, *Haemophilus influenzae *type b, other identified bacteria and bacterial meningitis of unknown aetiology.

The incidence of all age groups increased significantly in 1999, with the exception of small children, where there was a peak in 1998 (16.8/100 000 population), with a subsequent decrease. The group 1–5 year olds showed the highest incidence of all. (Table 1)

An overall and by age group significant predominance of male (63.2%) over female (46.8%) was observed in all the groups (data not shown).

Spn, Hib and *N. meningitidis *were the main causal pathogens identified.

In 1998 Hib showed the highest incidence (3.0 per 100 000 population), but dropped significantly by approximately half (1.6 per 100 000 population) after the vaccination programme in 1999 with a conjugated vaccine (Vaxem-Hib^®^). The greatest decrease of incidence was observed in small children, from 10.7 per 100 000 population in 1999 to 5.7 per 100 000 population in 2000 (p < 0.05). Only 8 cases have been reported since 2002 (Table 2).

**Table 2:** Overall and by age group Incidence and Case-Fatality Rate (CFR) of bacterial meningitis according to the aetiology. Cuba. 1998–2003.

***Streptococcus pneumoniae***						
**Age group**	**1998**	**1999**	**2000**	**2001**	**2002**	**2003**

1–5 yrs	1.6	2.8	2.9	2.0	1.9	2.3
6–11 yrs	0.3	0.9	0.9	0.6	0.2	0.7
12–14 yrs	0.4	0.6	1.1	0.5	0.4	0.7
15–18 yrs	1.4	0.8	0.8	0.3	0.3	0

**Overall Incidence**	**0.9**	**1.3**	**1.4**	**0.9**	**0.7**	**1.0**

**Overall CFR**	**20.0**	**13.1**	**28. 6**	**26.9**	**15.0**	**30.0**

***Neisseria meningitidis***						

**Age group**	**1998**	**1999**	**2000**	**2001**	**2002**	**2003**

1–5 yrs	1. 1	0.8	0.9	1.2	0.7	0.5
6–11 yrs	0.5	0.5	0.5	0.4	0.3	0.3
12–14 yrs	0.2	0.8	0.6	0.7	0.9	0.4
15–18 yrs	0.5	0.6	0.9	0.1	0.1	0.1

**Overall Incidence**	**0.6**	**0.6**	**0.7**	**0.6**	**0.5**	**0.3**

**Overall CFR**	**0**	**10.5**	**4.7**	**0**	**7.1**	**0**

***H. influenzae *type b**						

**Age group**	**1998**	**1999**	**2000**	**2001**	**2002**	**2003**

1–5 yrs	10.7	5.7	1.6	0.5	0.6	0
6–11 yrs	0.7	0.4	0.2	0	0	0.1
12–14 yrs	0	0.2	0	0	0	0.2
15–18 yrs	0	0	0	0	0	0

**Overall Incidence**	**3.0**	**1.6**	**0.5**	**0.1**	**0.2**	**0.1**

**Overall CFR**	**8.0**	**10.6**	**14.3**	**75.0**	**0**	**0**

**Unknown aetiology**						

**Age group**	**1998**	**1999**	**2000**	**2001**	**2002**	**2003**

1–5 yrs	4.6	6.2	4.7	4.3	1.3	2.2
6–11 yrs	2.7	5.6	2.1	2.6	1.9	2.8
12–14 yrs	1.2	3.4	1.1	2.2	3.0	2.1
15–18 yrs	1.7	3.0	3.4	2.3	1.2	1.7

**Overall Incidence**	**2.7**	**4.8**	**2.9**	**2.9**	**1.8**	**2.3**

**Overall CFR**	**7.7**	**2.9**	**5.9**	**7.1**	**9.6**	**7.8**

The overall incidence of pneumococcal meningitis was nearly 1 per 100 000 population, but a significant increase occurred in 1999 and 2000 (1.3 and 1.4 per 100 000 population respectively) and subsequently decreasing. A similar pattern in the incidence was observed in all age groups with the exception of adolescents from 15–18 years old. The small children showed the highest incidence of all groups, mainly in 1998 (2.8 per 100 000 population) and in 1999 (2.9 per 100 000 population), decreasing in 2001 and 2002, with a discreet subsequent increase in 2003 (2.3 per 100 000 population). Since the year 2000 Spn has shown the highest incidence of all the identified bacteria (Table 2).

*Neisseria meningitidis *was one of the three main causal pathogens of BM, but the overall incidence remained below 0.8 per 100 000 population, decreasing significantly towards 2003. All the age groups showed subtle variations of the incidence throughout the study period, however only small children exceeded 1 per 100 000 population (Table 2). The overall ratio of meningitis/septicaemia for all groups was 3 per 1 (Data not shown). *Neisseria meningitis *serogroup B was the only one isolated throughout the study in the Neisseria Laboratory at the Institute "Pedro Kourí" (IPK).

Regarding other identified bacteria, *Staphylococcus sp*., *Streptococci *(different from *S. pneumoniae*), *E. coli *and *Salmonella *were the most common pathogens. The frequency of this group of bacteria was 43% in small children, 28% in the older children, 13% in adolescents from 12–14 year olds and 16% in adolescents from 15–18 year olds. Gram-positive bacteria (63%) was significantly more frequent than the gram-negative (37%) (Data not shown).

The average CFR of BM was nearly 10.5%. Overall CFR showed variations, increasing significantly in 2000 (13.6%) and in 2001 (13.6%) when compared to 1998 (7.8%), with a subsequent significant decrease in 2003 (10.9%) (Table 1).

The most lethal pathogen was Spn with an average CFR = 27% (13–30%), followed by Hib with CFR = 10.7% (8–75%) and Meningococcus B with CFR = 4% (4.7–10.5%). The highest CFR (75%) observed in the period was caused by Hib in 2001, but no cases have been reported subsequently (Table 2).

Bacterial meningitis of unknown aetiology accounted for 500 cases throughout the period. The overall incidence showed a significant increase when compared in 1998 (2.7 per 100 000 population) to 1999 (4.8 per 100 000 population), as well as by age group. Only among small children a significant decrease was observed, from 4.6 per 100 000 population in 1998 to 2.2 per 100 000 population in 2003 (Table 2).

**Figure 1:**
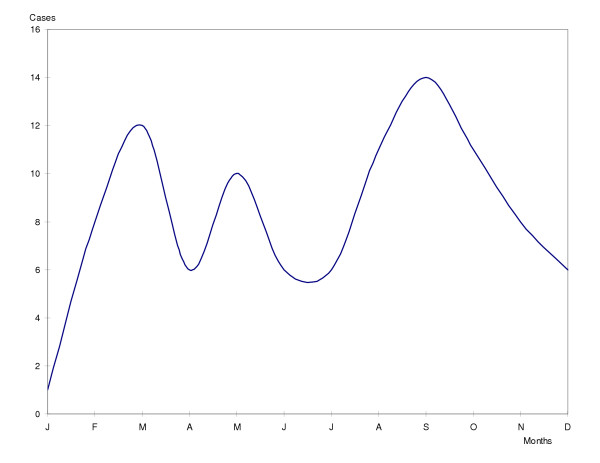
Seasonality of meningococcal meningitis in children and adolescents. Cuba. 1998–2003.

No seasonality was observed in pneumococcal and Hib meningitis. Only meningococcal meningitis showed seasonal behaviour with the highest peaks in September, October and March (Figure 1).

Fifteen percent of overall BM cases slept in overcrowded dormitories. This condition was observed in 10.1% of children, and increased significantly in adolescents from 12–14 year olds and 15–18 year olds (30.6% and 24.6% respectively) (Data not shown).

No association of BM with attendance to DCC or boarding school (including college, technical or professional education) was observed during the study. Only in 1998 and 1999 did the attendance to boarding high school show a strong association with disease (RR = 2.1; p > 0.05) (Table 3).

**Table 3:** Associations of bacterial meningitis with attendance to day care centres or boarding school. Cuba. 1998–2003.

**Year**	**Day Care Centres**		**Boarding High School**		**Boarding College, Technical or Professional Education**	
	
	**RR**	**CI**	**RR**	**CI**	**RR**	**CI**
1998	0.7	0.45–1.14	2.1	0.64–7.42	1.0	0.33–2.95
1999	1.3	0.84–2.02	2.1	1.02–4.50	1.0	0.42–2.68
2000	1.0	0.62–1.80	0.5	0.20–2.31	0.6	0.26–1.43
2001	0.4*	0.17–0.89	1.6	0.62–4.28	0.1*	0.03–0.68
2002	1.2	0.54–2.62	1.7	0.69–4.17	0.2*	0.04–1.03
2003	0.7	0.32–1.62	0.6	0.18–2.07	0.4	0.08–1.76

*p < 0.05 (chi-squared test or Fisher exact test as appropriate)

RR: Relative Risk

CI: Confidence Interval

## Discussion

Prevention and control of infectious diseases, especially among children and adolescents, has been a big concern for the Public Health Ministry (PHM) in Cuba. Hitherto vaccines are the most promising prospect for the prevention of community acquired BM. Therefore widespread use of effective vaccines may induce a major impact in preventing the disease, especially when they are used during a long period of time as a part of the NIP, as occurred in Cuba, where a decreasing trend of BM has been documented after vaccination against meningococcus serogroup B and C, and more recently, Hib [4,9,10,12].

Cuban strategy to prevent these infections has been an initial vaccination campaign continued through the NIP.

The first intervention was carried out in 1979 with an A+C vaccine (Pasteur-Merieux) during the meningococcal disease epidemic caused mainly by serogroup C (50,0%) and B (34,3%). The target population was 3, 245 046 individuals from 3 months of age to 19 years old, considered at high risk of disease, achieving 78.2% vaccination coverage. This intervention decreased significantly the serogroup C (7.2%) in 1980, but was ineffective against serogroup B, therefore the incidence continued to rise, but having serogroup B as a predominant causal agent (78.4%) [4,12].

By the end of 1988, after having being evaluated in a double-blind, placebo-controlled trial, the Cuban anti-meningococcal BC vaccine (VA-MENGOC B-C^®^) was first administered to high risk age groups and subsequently to all the population from 3 months of age to 24 years of age (an estimated 2 million people or more) [4,9]. Since 1991 this vaccine has been included in the NIP for children of 3 months of life (2 doses, 2 months apart), decreasing the nation-wide incidence of meningococcal meningitis to below 1 per 100 000 population. For that reason, the contribution of meningococcal meningitis to the overall incidence of BM in Cuba is very small at present [13].

During this period, serogroup B *Neisseria meningitidis *(classified by a commercial sera Wellcome Diagnostic, England) has been the only one isolated bacteria and subsequently confirmed by the Neisseria Laboratory at the IPK, where the strain B4. P1. 19.15 has been the most frequently identified, using ELISA [14]. It is likely due to the fact that VA-MEMGOC B-C prevents the disease, but does not eliminate the nasopharyngeal carriage [9].

Shifts in the frequency and circulation of each bacteria and/or strain into a region, country or continent depends on the interaction of both the pathogen and the host within settled environmental conditions, nevertheless important changes may be induced by prolonged and massive vaccination programmes.

Small children are widely considered a high-risk group for BM, as it was confirmed in our study, where the highest incidence was observed in less than 6 year olds. [15,16]. In Cuba Hib and Spn were the leading pathogens of BM in this group since 1993, as a result of previous nationwide vaccination against meningococcus [5,12].

Due to the increase of infant mortality caused by Hib infections, the PHM decided to start using an available conjugate vaccine against Hib in 1999. The vaccine was initially used in a campaign for small children and continued through the NIP [10]. The incidence of Hib meningitis dropped by 47% after initial vaccination, especially in small children, coinciding with reports in other countries where those vaccines had also been used. Another beneficial effect of conjugated Hib vaccine is the elimination of carriage among vaccinated populations [17-19].

For these reasons, we considered that the vaccination against Hib contributed decisively to the overall decrease of BM in small children described above, and undoubtedly the development of such vaccines has been one of the most important events in the history of the prevention and control of infectious diseases in paediatrics [1,20].

Spn is not a common cause of meningitis epidemics and outbreaks [21]. We considered that the steady low incidence of meningococcal meningitis as well as the drastic and significant reduction of Hib meningitis in small children achieved during 1999 in Cuba, may have contributed to an increase of pneumococcal meningitis in 1999 and 2000, especially in children under 6 years old. We hypothesised that Spn might have filled somehow the ecological niche laid down abruptly by Hib after the incidence decrease subsequent to vaccination. Since then Spn is the leading cause of BM in Cuba as also occurs in many regions of the world [22].

The improvement of the BM surveillance system in Cuba increases substantially quantitative and qualitative information about this group of infections [12]. Despite these achievements, an important number of cases still remains of unknown aetiology due to previous antibiotic therapy.

Regarding the gender, we observed in our study that infections in male predominated those in females, contrarily to other author that point out non significant differences between males and females [1].

CFR depends on, among other factors, the host response, the virulence of the pathogen and the quality and timeliness of medical attention. Average CFR was nearly 10% and similar figures are reported in many developed countries, where it could be as low as 2% and as high as 25% [23,24], though in developing countries it may be higher. Pneumococcus was the most lethal germ, with CFR nearly 25%, and similar figures are reported in other regions of the world [16,25,26].

Two well-defined seasons are recognised in Cuba: the rainy (April to September) and the dry (October to March). The monthly average case distribution of Hib and pneumococcal meningitis, did not show seasonality differences, but meningococcal meningitis did, increasing the number of cases mainly in September, October and March, coinciding with reports of the literature [3]. In Cuba, September and October is the transitional period of the rainy season to the dry, but also it is the end of the summer vacations and the start of the school year, when many children and adolescents share the same environment (DCC and schools) and are in close contact with one another. Coincidence of these climatic, environmental and socio-cultural conditions, undoubtedly may contribute to the seasonal increase of meningococcal meningitis in our country.

Airborne transmission of infections can be favoured by some environmental conditions such as attendance at boarding institutions, overcrowding areas, poor ventilation and intimate contact [3,15,27].

In our study we observed that the overall percentage of cases sleeping in overcrowded dormitories was 15%, but in adolescents reached nearly 30%. It should be considered as an important risk for the transmission of BM in this group, and must call the attention of both, MINED and PHM, for the avoidance of these conditions, where possible, especially in DCC and boarding school.

On the other hand, as a nation-wide improvement of the Cuban educational system during the seventies, a huge number of boarding school (Colleges, Technical and Professional Education) were built nationwide. In these institutions the intimate contact and promiscuity among boarding students may increase airborne infections [3,15,27-29]. Nevertheless no strong association of BM with attendance to boarding school or DCC was observed, reinforcing the criteria of the beneficial effect of massive and systematic vaccination against two of the main BM causal pathogens.

## Conclusion

Surveillance is a key subject for the control and prevention of infectious diseases and provides information about main epidemiological and microbiological aspects to put in place correct strategies.

Massive vaccinations against meningococcus, and Hib have decreased overall BM incidence in Cuba, and showing Pneumococcus as the leading causal pathogen and the most lethal.

The disease was more frequently observed among male children under 6 years of age.

Seasonality was only observed in meningococcal meningitis. Dry season and the start of the school year may also be contributing environmental conditions.

There was a weak association of BM with attendance to DCC and boarding school, and may be the effect of massive and systematic vaccination, which in our opinion are the most adequate and effective measures for the prevention and control of these infections.

Further massive vaccination initiatives with available anti-pneumococcal conjugated vaccine (when economically possible in Cuba) may decrease the incidence of BM even more. Surveillance systems will contribute towards the detection of the emergence of other causal pathogens or any other changes in the epidemiology of these infections.

## Competing interests

The author(s) declare that they have no competing interests.

## Authors' contributions

FDM conceived the study, participated in the design, performed the statistical analysis, and the draft of the manuscript and was the main responsible author.

APR participated in the design and draft of the manuscript. All authors read and approved the final manuscript.

## Pre-publication history

The pre-publication history for this paper can be accessed here:


